# Does occupational forward bending of the back increase long-term sickness absence risk? A 4-year prospective register-based study using device-measured compositional data analysis

**DOI:** 10.5271/sjweh.4047

**Published:** 2022-10-29

**Authors:** Nidhi Gupta, Søren Skotte Bjerregaard, Liyun Yang, Mikael Forsman, Charlotte Lund Rasmussen, Charlotte Diana Nørregaard Rasmussen, Els Clays, Andreas Holtermann

**Affiliations:** 1 Department of musculoskeletal disorders and physical workload, National Research Centre for the Working Environment, Copenhagen, Denmark.; 2 Department of analysis and data, National Research Centre for the Working Environment, Copenhagen, Denmark.; 3 School of Engineering Sciences in Chemistry, Biotechnology and Health, KTH Royal Institute of Technology, Huddinge, Sweden.; 4 IMM Institute of Environmental Medicine, Karolinska Institutet, Stockholm, Sweden.; 5 Department of Public Health and Nursing, Norwegian University of Science and Technology, Trondheim, Norway; 6 Faculty of Physical Culture, Palacký University, Olomouc, Czech Republic.; 7 Department of Public Health and Primary Care, Ghent University, Ghent, Belgium.; 8 Department of Sports Science and Clinical Biomechanics, University of Southern Denmark, Odense, Denmark.

**Keywords:** occupational activity, occupational health, sick leave, trunk flexion

## Abstract

**Objective:**

Forward bending of the back is common in many jobs and a risk factor for sickness absence. However, this knowledge is based on self-reported forward bending that is generally imprecise. Thus, we aimed to investigate the dose–response relation between device-measured forward bending at work and prospective register-based risk of long-term sickness absence (LTSA).

**Methods:**

At baseline, 944 workers (93% from blue-collar jobs) wore accelerometers on their upper back and thigh over 1–6 workdays to measure worktime with forward bending (>30˚ and >60˚) and body positions. The first event of LTSA (≥6 consecutive weeks) over a 4-year follow-up were retrieved from a national register. Compositional Cox proportional hazard analyses were used to model the association between worktime with forward bending of the back in an upright body position and LTSA adjusted for age, sex, body mass index (BMI), occupational lifting/carrying, type of work, and, in an additional step, for leisure time physical activity (PA) on workdays.

**Results:**

During a mean worktime of 457 minutes/day, the workers on average spent 40 and 10 minutes on forward bending >30˚ and >60˚ in the upright position, respectively. Five more minutes forward bending >30˚ and >60˚ at work were associated with a 4% [95% confidence interval (CI) 1.01–1.07] and 8% (95% CI 1.01–1.16) higher LTSA risk, respectively. Adjustment for leisure-time PA did not influence the results.

**Conclusion:**

We found a dose–response association between device-measured forward bending of the back and prospective LTSA risk. This knowledge can be integrated into available feasible methods to measure forward bending of the back for improved workplace risk assessment and prevention.

High ergonomic work demands are established risk factors for long-term sickness absence (LTSA) (1), and LTSA puts a large burden on the workplaces and society (2). Thus, many workplaces aim to prevent LTSA by assessing and intervening on the ergonomic work demands (3–7).

Forward bending of the back while in an upright position (when workers are on their feet) is a prevalent ergonomic exposure (8) and associated with an increased risk of sickness absence (9, 10). However, this knowledge is either based on self-reports, workplace observations, or expert opinions, all with major limitations. Self-reported forward bending at work is shown to correspond poorly with device-based measurements (11). Moreover, both workplace observations and expert opinions of body movements such as forward bending are known to be of low precision, or only based on a short period of the worktime (12). Thus, existing knowledge about the dose–response relation of forward bending at work with sickness absence risk can at best be imprecise and, at worst, biased.

To obtain valid measurements of forward bending at work, feasible device-based (eg, accelerometers) measurement systems are now available. These systems can accurately measure forward bending at work over several days (13). Recent research has focused on investigating the dose–response relation between device-measured worktime spent forward bending of the back at various degrees (eg, >30˚ and >60˚) and musculoskeletal pain (14). However, work environment researchers, practitioners, and stakeholders lack knowledge on the dose–response relation between device-measured forward bending at work and sickness absence risk. Such knowledge is warranted for better risk assessment and preventive initiatives.

We aimed to investigate the dose–response relation between worktime with forward bending of the back in an upright position and the prospective risk of LTSA.

## Methods

### Study design and population

This was a four-year prospective study where individuals were invited to participate in baseline measurements. Baseline measurements included accelerometry measurements, a questionnaire, and a health check. Individuals were then followed up in the national register to obtain information on the first event of LTSA within four years following the date of baseline (ie, from the last day of accelerometry measurement). This meant that each worker had an equal follow-up period of four years (ie, 212 weeks).

This study used baseline data from the ‘Physical wOrk DEmands and Prospective register-based Sickness Absence’ (PODESA) study (15, 16) and prospective data on sickness absence from the national register. PODESA consists of harmonized data from the ‘New method for Objective Measurements of physical Activity in Daily living’ (NOMAD) (17) and the ‘Danish PHysical ACTivity cohort with Objective measurements’ (DPhacto) (18) cohorts. In both cohorts, labor unions assisted in recruiting participants from 22 workplaces within the manufacturing, cleaning, transport, healthcare, garbage collection, construction, assembling, and mobile plant operations sectors in Denmark. All workers from these workplaces were invited to local information meetings where study details were provided and were offered participation. Of the 2107 workers in DPhacto and 391 workers in NOMAD who were offered participation, 1390 (55.6%) workers either participated in the questionnaire and/or health check at the baseline. The baseline data in the NOMAD and DPhacto cohorts was collected from 2011–2012 and 2012–2013, respectively. Previous studies on DPhacto found no relevant differences between participants and non-participants at baseline for the demographics and lifestyle-related factors (18). Similarly we found no relevant differences in NOMAD cohorts between those who wanted and did not want to participate [non-participants (N=88) age: 43.4 years, females: 34%, job seniority: 151 months, daily smokers: 37%; participants (N=262) age: 44.6 years, females: 40%, job seniority: 165 months, daily smokers: 34%). More details on how we recruited these workplaces and harmonized these cohorts and their background information are provided in our previously published articles (15, 18) and more details on the flow of the participants are given in the supplementary material (www.sjweh.fi/article/4047), appendix A.

### Ethical approval

The Ethics Committee for the Capital Region of Denmark approved the DPhacto and NOMAD cohorts (file number H-2-2012-011 and H-2-2011-047) (15). All eligible workers received written and oral information about (i) the practicalities of participation, (ii) potential risks of participating, and (iii) freedom of withdrawing from the project. Individuals provided written consent to participate in the study and to use their data for research purposes.

### Accelerometry

In the PODESA cohorts, participants were offered to wear Actigraph (GTX3+, Florida, USA.) accelerometers on the right arm, right thigh, upper back, and hip for 4–7 consecutive workdays including at least two workdays (19, 20). This study used data from the right thigh and upper back to measure forward bending of the back while in the upright position. For a sensitivity analysis (refer to statistical analyses section), we also used data from the arm accelerometer to get information on elevated arm above shoulder height (used as a confounder). During the measurement period, workers were also asked to fill out a short daily diary indicating the time of starting and ending their primary work and time of getting in and out of the bed on each measured day.

The accelerometer data were downloaded using the ActiLife Software version 5.5 and further processed using the valid MATLAB program Acti4 (13, 20, 21). First, using the thigh-based accelerometer data and the self-reported diary information, we determined worktime spent in the upright position (ie, time with standing, walking, running, and stair climbing) and non-upright position (ie, sedentary as sitting or lying). Second, data from the upper back accelerometer were used together with data from the thigh-based accelerometer to determine how much of the worktime in the upright position was spent with forward bending of the back at ≤30˚, >30˚, ≤60˚, and >60˚. We chose these cut points because of their previously shown relevance for musculoskeletal pain (14, 22, 23).

A workday was defined as a 24-hour day when the participant is working. A work period was defined as a continuous period/work shift when the participant spends time on their occupation. A participant could have several work periods within one workday (for example, an industrial manufacturing worker is working 00.00–07.00 and then 19.00–00.00 hours). Hours spent in all work periods within one workday were summed together to calculate total working hours per day.

Data for each participant on all postures and movements were averaged across all valid measured work periods. A work period for a worker was considered valid if consisted of ≥4 work hours or ≥75% of the average measured work time/day for that worker. In the analyses, we included all workers with at least one valid work period (average measured work period range: 5–95^th^ percentile 5.2–10.5 hours or 312–630 minutes).

### Register-based long-term sickness absence (LTSA)

We retrieved information on the first event of LTSA during the four-year follow-up from baseline via the Register-based Evaluation of Marginalization (DREAM) register (24) using the workers’ unique civil registration number. The DREAM register contains information on granted subsidized sickness absence benefits per week given by the state to the workplace of each sick worker in Denmark. DREAM register contains around 100 codes for various social transfers. The study by Madsen & Larsen (25) describes which of these codes to be used when determining sickness absence based on the DREAM register. These sickness absence benefits are provided after 30 continuous days of sickness absence. Thus, we chose ≥6 continuous weeks as the cut point to be certain that we captured all sickness absence events lasting >30 continuous days of sickness absence. This is also the reason why other studies from Denmark using the DREAM register to define LTSA have used the cut point of ≥6 continuous weeks (1, 26).

### Potential confounders

We chose the potential confounders a priori based on previous evidence on the association of ergonomic exposures, including forward bending at work, with musculoskeletal pain and sickness absence (9, 27, 28). We determined the age of the workers using their unique civil registration number while we identified the sex of the workers using a single item ‘Are you male or female’? We measured the body mass index (BMI) of the workers by objectively measuring their weight (kg) and height (cm). We determined how much time the workers spent on lifting and carrying at work via a single item with 6 responses ranging from ‘almost all the time’ to ‘never’ (29). We retrieved information on the type of work using a single item “Are you a worker engaged in administrative work tasks (white-collar) or production (blue-collar)?” as a proxy of socioeconomic status (30). We identified the event of LTSA within 12 months before baseline using the DREAM register. We determined how much influence workers had at work using two items from the Copenhagen Psychosocial Questionnaire (31, 32). The two items for influence at work were “Do you have a large degree of influence concerning your work?”; “Can you influence the amount of work assigned to you?”. Scores on these items were summarized separately into a scale of 0 to 100% where 0 meant no influence at work. We measured leisure time physical activity (time spent on sedentary behavior and upright position) using thigh accelerometry (16).

### Statistical analysis

Measured data on worktime with forward bending of the back is compositional (33). Composition of various exposures in worktime, ie, time with forward bending of various degrees while in the upright position and total time with non-upright position, for each participant sums up to 100% of worktime. Additionally, these exposures in the worktime composition are inherently co-dependent. The traditional analytical approach is not designed to handle such compositional data. Accordingly, we followed a compositional data analysis (CoDA) approach to perform all statistical analyses. For a thorough understanding of the implementation of CoDA in occupational research, please read the explanation here (33, 34).

**Figure 1:**
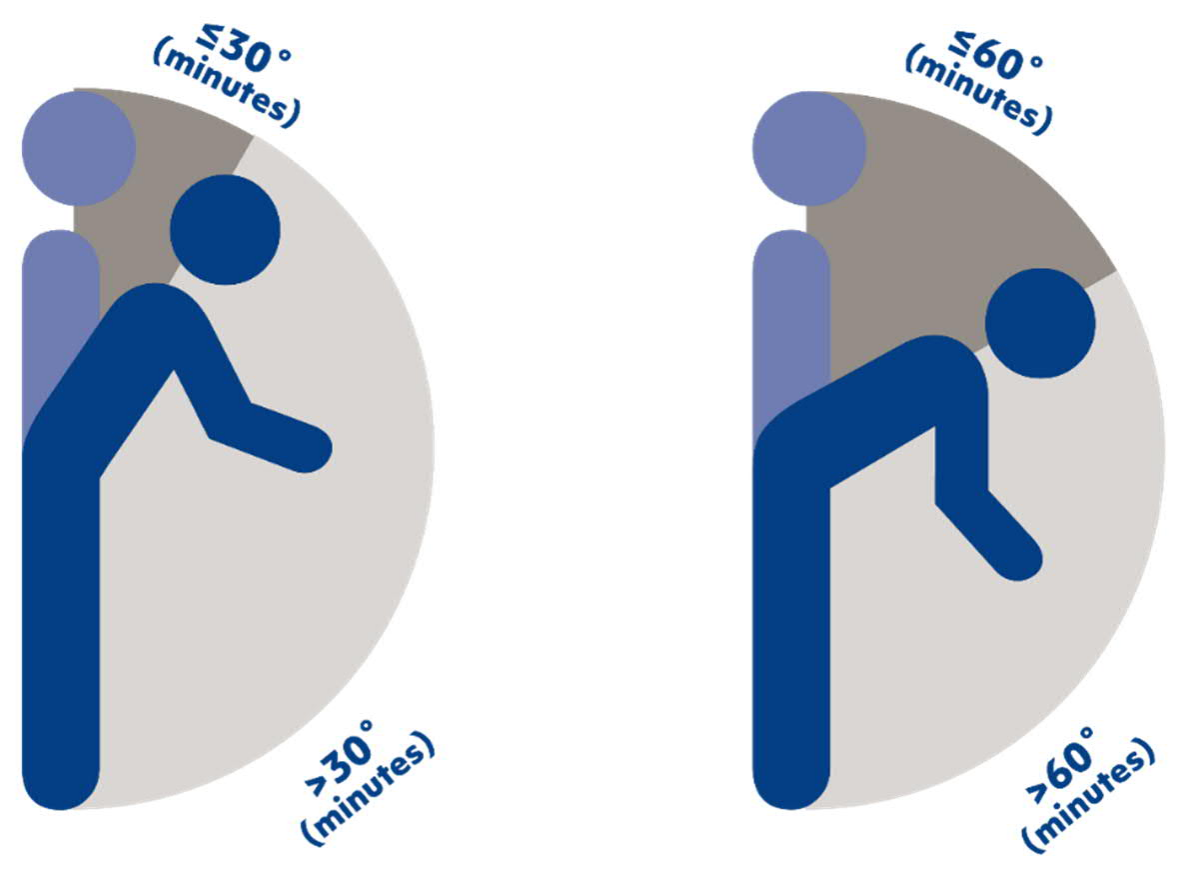
Illustration of the two worktime compositions of forward bending in an upright position used in analyzing the association between worktime with forward bending of the back at >30˚ (A) and >60˚ (B) and risk of long-term sickness absence. Please note that the worktime with non-upright (sitting or lying) position is also part of the worktime composition used in the statistical analyses, although not visualized in the figure.

For the analyses, we first defined two types of compositional exposures (figure 1):

- “Composition A” consisted of three exposures: (i) worktime with forward bending of the back >30˚ in the upright body position, (ii) worktime with forward bending of the back ≤30˚ in the upright body position, and (iii) worktime in the non-upright body position (figure 1A).
- “Composition B” consisted of three exposures: (i) worktime with forward bending of the back >60˚ in the upright body position, (ii) worktime with forward bending of the back ≤60˚ in the upright body position, and (iii) worktime in the non-upright body position (figure IB).

### Main analyses

The main analyses consisted of three steps: Step 1) we transformed each composition (ie, worktime composition A or B) to isometric log-ratios [ilrs, see these articles (34, 35) to understand how ilrs are calculated and interpreted]. For each composition, this transformation resulted in two ilrs (ilr1 and ilr2). These are the following equations for calculating ilr1 and ilr2 for Composition B.


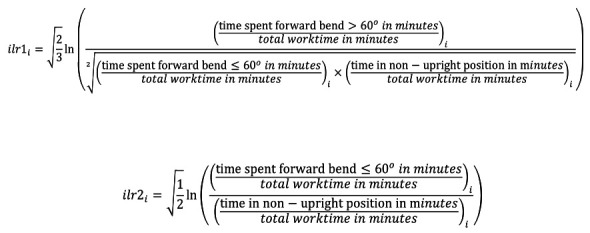


Where *i* is one worker.

Step 2) we performed two separate Cox proportional hazards regressions (one for each composition), modeling both ilrs against the onset of LTSA event (34). The models were adjusted for age, sex, BMI, worktime with lifting/carrying, and type of work (blue-collar or white-collar). Ilr1, ilr2, age, BMI, and lift/carry duration at work were modeled as continuous variables while the remaining were modeled as categorical variables. The resulting cox model and its interpretation is given in the supplementary material of Gupta & Rasmussen (34) article.

In the Cox models, each worker contributed with risk time until the first event of LTSA occurred or until the end of a 4-year follow-up in case of no event. During the 4-year follow-up, 45 workers dropped out for one of the following reasons: emigrated, died, entered early retirement, entered ordinary retirement, or became pregnant. These workers contributed to the risk time in the analyses until the week of dropping out.

We verified the assumption of the proportional hazards via visual inspection and the Grambsch-Therneau test (36). We assessed the statistical significance of the association between worktime compositions and LTSA risk using the Type-II likelihood-ratio tests. We considered the results to be significant at P<0.05.

Step 3) the regression coefficients of the ilrs (the effect sizes) obtained from the Cox models were in logarithmic scale, which were difficult to interpret (see these coefficients in supplementary appendix C). Thus, we used compositional isotemporal substitution analysis to interpret these logarithmic coefficients (29, 34).

Briefly, we first calculated a ‘reference composition’ ie, the sample mean worktime with forward bending while in the upright position and non-upright position. For example, for composition A, this was 40 minutes >30˚, 261 minutes ≤30˚, and 156 non-upright minutes (table 2). Based on the reference composition, we calculated new theoretical compositions by incrementally reallocating a fixed amount of time from one exposure to another exposure of the composition while keeping the time in the non-upright position and total worktime constant. For example, for composition A, we reallocated 5 minutes of forward bending >30˚ to forward bending ≤30˚ keeping constant the non-upright time (156 minutes) and the total worktime (457 minutes or 7.6 hours). This resulted in the following new theoretical composition: 35 minutes >30˚, 266 minutes ≤30˚, and 156 non-upright minutes. We performed similar reallocations for both compositions (A and B). The size of the reallocations was chosen to keep the resulting theoretical compositions within the range of the measured exposures.

**Table 2:** Compositional means of the measured worktime per day with forward bending of the back >30° (Composition A) and >60° (Composition B) among workers without (N=740) and with (N=204) an event of long-term sickness absence (LTSA) and among the total population (N=944).

Variables	Without event (N=740)			With event (N=204)			Total (N=944)		
		
	N	% ^a^	Mean (mins)	N	% ^a^	Mean (mins)	N	% ^a^	Mean (mins)
Composition A									
≤30	740	53	262	204	52	258	944	57	261
>30°	740	13	39	204	15	45	944	9	40
Non-upright position ^b^	740	34	157	204	33	150	944	34	156
Composition B									
≤60	740	64	292	204	64	292	944	64	291
>60°	740	2	9	204	2	11	944	2	10
Non-upright position ^b^	740	34	157	204	33	150	944	34	156

a % represents that these exposures of the worktime composition are presented as the proportion of total measured worktime per day (table 1).

b Non-upright position includes sit and lie postures.

The new theoretical compositions were then transformed to ilrs using the formulas given above. Using the regression coefficients obtained from the Cox models (shown in supplementary appendix C), we predicted hazards ratios (HR) and their 95% confidence intervals (CI) for these theoretical ilrs indicating the predicted difference in the LTSA risk corresponding to the difference between the new theoretical composition and the reference composition. Hence, the predicted HR indicated the relative risk of LTSA attributed to a higher/lower duration of forward bending. The formula for predicting the HR and their 95% CI is given in the supplementary file of the Gupta & Rasmussen (34) article.

Finally, we plotted these predicted HR together with their 95% CI on the y-axis against measured worktime spent on forward bending >30° or >60° (in minutes) on the x-axis.

To interpret the results in terms of absolute risk of LTSA, we rewrote the Cox model as S(*t*\*X*)=S(*t*)^exp(*X*β)^, where S(*t*\*X*) is the probability of survival (no LTSA-event) past time *t* given the values of the predictors *X* (ilrs and confounders in our case), and *β* is the regression coefficients. Based on the Cox model estimates, we estimated Kalbfleisch-Prentice-Cox survival function S(*t*\*X*), which is adjusted for the predictors (ilrs and confounders). The effect of the predictors is to raise the survival function to power of exp(*X*β)(37). We then plotted the predicted cumulative risk of LTSA F^(*t*\*X*)=1−S^(*t*)^exp(*X*β^)^ for varying time reallocations of forward bending to make the results interpretable. is a cumulative risk at a certain time point ‘t’.

### Sensitivity analyses

To test the sensitivity of the results obtained from the main analyses, we also performed these additional analyses: (i) Due to technical errors, some workers could not answer questions on influence at work. Thus, the main analyses were performed without and with additional adjustment for influence at work for the remaining 739 workers; (ii) We also performed a separate analysis where we excluded the workers who had pre-events of LTSA, ie, events within 12 months before baseline (N=57); (iii) We performed the main analyses with and without adjustment for leisure time physical activity on workdays (N=828, average leisure time=476 minutes, 5–95^th^ percentile=302–697 minutes or 5.04–11.6 hours); (iv) The observed association in the main analysis could be confounded by other “co-occurring” ergonomic exposures, such as work with elevated arm above shoulder height. Thus, we performed a sensitivity analysis where we also adjusted the statistical model for minutes of worktime spent with elevated arm above shoulder height in the upright position (N=924), measured using arm-based and thigh-based accelerometry as described previously (34).

All analyses were performed in the R software (version [3.5.1]) using the software packages ‘Compositions’ (38), ‘robCompositions’ (39) and ‘survival’ (40).

## Results

### Participant flow and descriptives

Of the 2498 eligible and invited workers, 944 were included in the main analysis as they had valid data on at least one working period and provided their unique civil registration number to obtain information on LTSA from the DREAM register. More details on the participant flow are given in supplementary appendix A.

These 944 workers were, on average, 45 [standard deviation (SD) 9.7] years old and had an average BMI of 27.2 kg/m^2^ (SD 4.9). Additionally, 43.9% of them were women, 93.2% of them were engaged in blue-collar occupations, and 58.9% were working in the manufacturing sector. In total, 204 workers (21.6%) had their first event of LTSA at 78th week (median) within the 4-year (ie, 212 weeks) follow-up time. The analyses included a total of 167,184 person-years during the follow-up time.

Workers were, on average, measured for 3 days with a range of 1–6 workdays. The variation in the measured number of days between workers was due to differences in measured non-workdays, sick days, vacation days, and non-valid work periods (A work period for a worker was considered valid if consisted of ≥4 work hours or ≥75% of the average measured work time/day for that worker). On average, the whole analytical sample was measured for 457 minutes (7.6 hours) of worktime/day, of which 156 minutes were spent in a non-upright body position (sitting or lying) and 301 minutes were spent in the upright body position. Of these 301 minutes spent in the upright body position; in Composition A, 40 minutes were spent on forward bending >30˚ and 261 minutes on forward bending ≤30˚. In composition B, 10 minutes were spent on forward bending >60˚ and the remaining 291 minutes on forward bending ≤60˚. We have added a ternary plot to visualize the distribution of the forward bending of the back at >30˚and ≤30˚, and at >60˚ and ≤60˚ (supplementary appendix B).

Comparing workers without (N=740) and with (N=204) LTSA event, no major differences in baseline descriptive characteristics were found, except that the group with LTSA event had relatively more women, had slightly less influence at work and had more pre-events of LTSA (ie, LTSA event during 12 months before baseline) (see table 1).

**Table 1:** The baseline descriptive of the workers without (N=740) and with (N=204) an event of long-term sickness absence (LTSA) and of the total population (N=944).

Variables	Without event (N=740)			With event (N=204)			Total (N=944)		
		
	N	%	Mean (SD)	N	%	Mean (SD)	N	%	Mean (SD)
Age (years)	740		45.0 (9.7)	204		43.8 (10.3)	944		44.7 (9.9)
Women	298	40.3		116	56.9		414	43.9	
BMI (kg/m^2^)	727		27.1 (4.8 )	202		27.2 (5.2)	929		27.2 (4.9)
Occupational lifting/carrying duration (1–6) ^a^	737		3.8 (1.4)	204		3.5 (1.5)	941		3.7 (1.4)
Influence at work (0–100%) ^b^	580		58.0 (28.3)	159		52.9 (29.9)	739		56.9 (28.7)
White-collar	55	7.4		9	4.4		64	6.8	
Blue-collar	685	92.6		195	95.6		880	93.2	
Job sector									
Cleaning	115	15.5		46	22.5		161	17.1	
Manufacturing	445	60.1		111	54.4		556	58.9	
Transport	59	8.0		20	9.8		79	8.4	
Health Service	11	1.5		8	3.9		19	2.0	
Assemblers	29	3.9		3	1.5		32	3.4	
Construction	31	4.2		7	3.4		38	4.0	
Garbage collectors	19	2.6		6	2.9		25	2.6	
Mobile plant operators and others ^c^	31	4.2		3	1.5		34	3.6	
Pre-event of LTSA	28	3.8		29	14.2		57	6.0	
Maternity	23	3.1		0	0		23	2.4	
Total measured worktime/day (minutes)	740	100	458 (92)	204	100	453 (82)	944	100	457 (90)
Total measured leisure time/day (minutes)	654	100	475 (114)	174	100	481 (112)	828	100	476 (114)
Total measured waking hours/day (minutes)	654	100	925 (113)	174	100	929 (107)	828	100	926 (112)

a 1=almost all the time, 6=never.

b 0% means no influence at work/no support at work.

C Other includes general office clerks, and other elementary workers.

### Main analysis

Results of the Compositional Cox Proportional Hazard models on the 944 workers showed a statistically significant association (using the likelihood ratio test) between worktime compositions of forward bending of the back ≤30˚ and >30˚ while in the upright position (composition A; χ2=7.0, P=0.03) and LTSA risk. Similar but borderline non-significant results were observed for worktime composition of forward bending of the back ≤60˚ and >60˚ (Composition B; χ2=5.8, P=0.06). The resulting estimates obtained from the models are presented in Appendix C.

**Figure 2:**
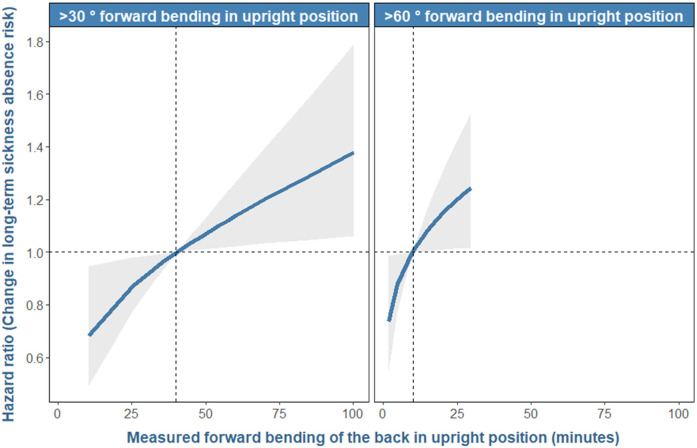
Results of the direction and strength of the association between worktime with forward bending of the back >30˚ and >60˚ in an upright position and prospective relative risk of long-term sick-ness absence among 944 workers. The X-axis represents the measured minutes of worktime spent with forward bending of the back >30˚ and >60˚. Y-axis shows the hazards ratio indicating the risk of long-term sickness absence relative to the risk associated with the “average” forward bending of the back. These averages for >30° and >60° were 40 and 10 minutes, respectively and are indicated by the vertical dotted line on the x-axis. The associated risk at these averages is 1 and is indicated by the horizontal dotted line on the y-axis. Refer to figure 3 for the absolute risk associated with these average time. The ribbons along the line show the 95% confidence intervals of the resulting estimates. The reason behind why we observe non-linear patterns in these figures is given in these articles (34, 50).

For composition A (ie, forward bending above and below 30˚), from the average, reallocating five more minutes to forward bending of the back >30˚ in the upright position per day was associated with a 4% (HR 1.04, 95% CI 1.01—1.07) higher risk of LTSA at any given time point in the 4-year follow-up period (figure 2). Please note that these extra minutes of forward bending of the back >30˚ were obtained by subtracting five minutes from forward bending ≤30˚, and keeping the remaining worktime (ie, non-upright) constant. Similarly, reallocating five more minutes to forward bending of the back >60˚ in the upright position was associated with an 8% (HR 1.08, 95% CI 1.01–1.16) higher LTSA risk (figure 2).

**Figure 3:**
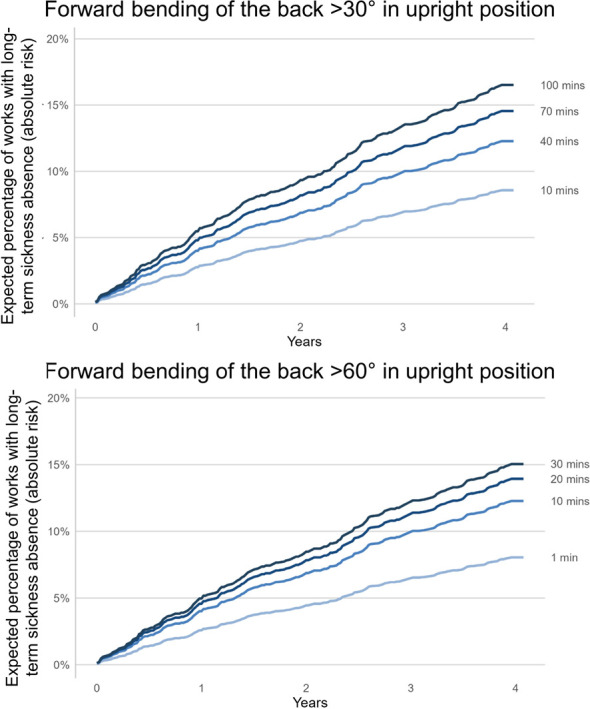
Results of the direction and strength of the association between worktime with forward bending of the back >30˚ and >60˚ in an upright position and prospective absolute (instead of relative risk as shown in Figure 2) risk of long-term sickness absence over the course of 4-year follow up among 944 workers. The Y-axis shows the proportion of workers with long-term sickness absence while the x-axis shows the follow-up time. Each solid line represents a certain amount of worktime with forward bending >30˚ and >60˚ and associated absolute risk of LTSA. From the estimated Kalbfleisch-Prentice-Cox survival function, absolute risk related to forward bending >30˚ and >60˚ was calculated at the grand mean of other predictors (age = 44.7; sex = men; body mass index = 27.2; occupational lift/carry duration = 3.7; type of work = blue-collar). See Methods section to understand how we have calculated the absolute risk.

Figure 3 displays the predicted absolute LTSA risk over time up to 4 years for worktime spent forward bending >30˚ (Composition A) and >60˚ (Composition B). The assumption was that the exposure, ie, time spent on forward bending, remained the same throughout the follow-up period. At the end of the fourth year, the risk of LTSA for 10 minutes forward bending >30˚ was 9%. This risk increased to 12.2% at 40 minutes forward bending >30˚ (representing the average of this population), and 14.5% at 70 minutes of forward bending >30˚. Similarly, 1, 10, and 30 minutes of forward bending >60˚ were associated with 8%, 12.3%, and 15% absolute risk of LTSA, respectively. The relative risk (HR) and absolute risk are related. For example, five minutes higher forward bending >30˚ and >60˚ from its average was associated with 0.4 and 1.0%-point increase in LTSA absolute risk and 4% and 8% relative risk at the grand mean of all remaining variables in the model (variables indicated in step 2 of the Statistical Analysis section), respectively.

### Sensitivity analyses

Overall, the results of the main analyses and the four sensitivity analyses were similar (results not shown). For example, when not adjusting for worktime with elevated arm above shoulder height in the upright position, we found that reallocating five more minutes to forward bending of the back >60˚ in an upright position was associated with an 8% (HR 1.08, 95% CI 1.00–1.16) higher LTSA risk. When we adjusted for worktime with elevated arm above shoulder height in the upright position in the statistical model, the corresponding results remain similar (HR 1.07, 95% CI 1.00–1.15).

## Discussion

As far as we know, our study is the first to investigate the dose–response relation between device-measured forward bending of the back at work and prospective register-based risk of LTSA.

Our finding that device-measured forward bending of the back at work increases the prospective risk of LTSA corroborates findings in previous studies using self-reported measures (41, 42). However, the main novelty of our study is that we can quantify the relationship with a resolution down to minutes spent on forward bending of the back and LTSA risk. We found that five more minutes forward bending >30˚ and >60˚ were associated with 4% and 8% higher risk, respectively. We consider this to be a big step forward compared to previous studies based on very gross exposure information on forward bending at work using self-reports, workplace observations, or expert ratings. For example, previous studies (10, 11) used questionnaires where workers reported their worktime spent on forward bending in the following categories; “never”, “sometimes”, “25%”, “50%”, “75%”, and “more than 75%” of the worktime. Based on the knowledge from device-measured forward bending at work, it is obvious that these gross exposure categories do not provide realistic information of the true exposure of forward bending, and not being precise enough to give a sufficient resolution of the exposure down to minutes (10, 11). Additionally, self-reported ergonomic exposures are shown to be systematically misclassified depending on factors such as musculoskeletal complaints and sex (43, 44). These limitations restrict the use of self-reports in risk assessment and designing effective workplace initiatives to prevent sickness absence. Our study uses accurate forward bending measurements (13) and provides quantifiable LTSA risk estimates for as little as 5 minutes of forward bending of the back. These specific estimates on the true work exposure are valuable for risk assessments and workplace interventions and thus important for occupational health professionals and practitioners. In our previous study (34), we published results on the association between worktime spent with elevated arms and LTSA risk using the similar measurement method used in this study. This means that practitioners can use the same method for risk assessment of both elevated arm work and work with forward bending of the back.

Our study also shows that the dose–response association between forward bending of the back and LTSA risk is steeper for >60˚ than >30˚ of forward bending. This finding can be explained by the higher load on the structures of the back with forward bending at >60˚ compared with >30˚ (45, 46), and suggests that particularly much worktime spent with forward bending of the back >60˚ should be reduced for preventing LTSA.

We believe that our study, providing more specific and valid estimates of the dose–response association between forward bending of the back and LTSA, generates valuable knowledge for practitioners, employers, and policymakers aiming to improve the prevention of LTSA due to high ergonomic exposures. For example, figure 3 shows the absolute LTSA risk of workers with different exposures of forward bending at work at 1, 2, and 4 years. We believe this knowledge can encourage practitioners and workplaces to use feasible measurement tools to perform an accurate risk assessment of forward bending of the back. If practitioners and workplaces are provided with such specific measures of the true exposure of forward bending at work, and its associated risk of LTSA, we believe that it can improve workplace interventions targeting forward bending of the back and prevention of LTSA.

To perform such accurate risk assessment and design specific workplace preventive interventions, workplaces can tap upon accessible and user-friendly device-based tools to measure ergonomic exposures. Examples of such tools that are available for researchers and practitioners can be found here (47–49). The prices of the ergonomic measurement devices are becoming lower and the device-based systems’ usability and feasibility is increasing. Thus, the integration of knowledge produced in this study into the feasible device-based tools will be the way forward for collecting large-scale data, performing accurate risk assessment, and performing better workplace prevention.

### Strength and limitations

One limitation was the inclusion of only 37% of the total sample in the main analyses. This was both due to workers not willing to participate or it was not feasible to perform measurements on workers due to vacation, travelling, sick leave, odd working periods or shortage of accelerometers etc. However, previous studies on the DPhacto cohort did not indicate any differences between those who participated and those who did not (17, 18). Similarly, we found no significant differences for the NOMAD cohort between non-participants and participants (see results presented in the section “Study design and population”). Another limitation was the lack of information on the “load” (eg, if the forward bending was performed while lifting heavy weights) when performing forward bending of the back. The potential occurrence of residual confounding and bias in observational non-randomized studies is a general limitation. The lack of information on the cause of sickness absence was another limitation of the study.

An obvious strength was the use of accelerometry to measure worktime with forward bending of the back in combination with body position. Another strength was the use of recommended CoDA-based analyses in handling compositional data like worktime with forward bending of the back. The application of a prospective-study design and the use of register-based LTSA were additional strengths of the study. An additional strength was also that we investigated if adjustment for a potential “co-occurring ergonomic work exposure” arm elevation above shoulder height would influence our results on the association between forward bending of the back and LTSA risk. We did not adjust for the worktime composition of arm elevation (that also includes time spent with arm elevation below shoulder level) in the analysis. This is because of the statistical challenges of modeling two potential overlapping compositions (of ergonomic work exposures such as forward bending of back and arm elevation) in the same model. In the future, we should develop analytical methods that can model the effect of two overlapping compositions of ergonomic exposures and their effect on sickness absence.

### Concluding remarks

We found a clear dose–response association between device-measured forward bending of the back and register-based prospective LTSA risk. Five more minutes worktime spent on forward bending of the back >30˚ and >60˚ were associated with 4% and 8% higher risk of LTSA, respectively. We consider this new knowledge of specific and realistic dose–response association between forward bending of the back at work and LTSA risk to be useful and valuable for workplace prevention practices by using new practical and feasible device-based tools.

## Supplementary material

Supplementary material
