# Supplementary material for: Does occupational forward bending of the back increase long-term sickness absence risk? A 4-year prospective register-based study using device-measured compositional data analysis
Source: Scand J Work Environ Health. 2022 Oct 29;48(8):651–61. doi: 10.5271/sjweh.4047 (PMC10546616; doi:10.5271/sjweh.4047)
Supplement: Supplementary material [file SJWEH-48-651-S001.pdf]

Supplementary material

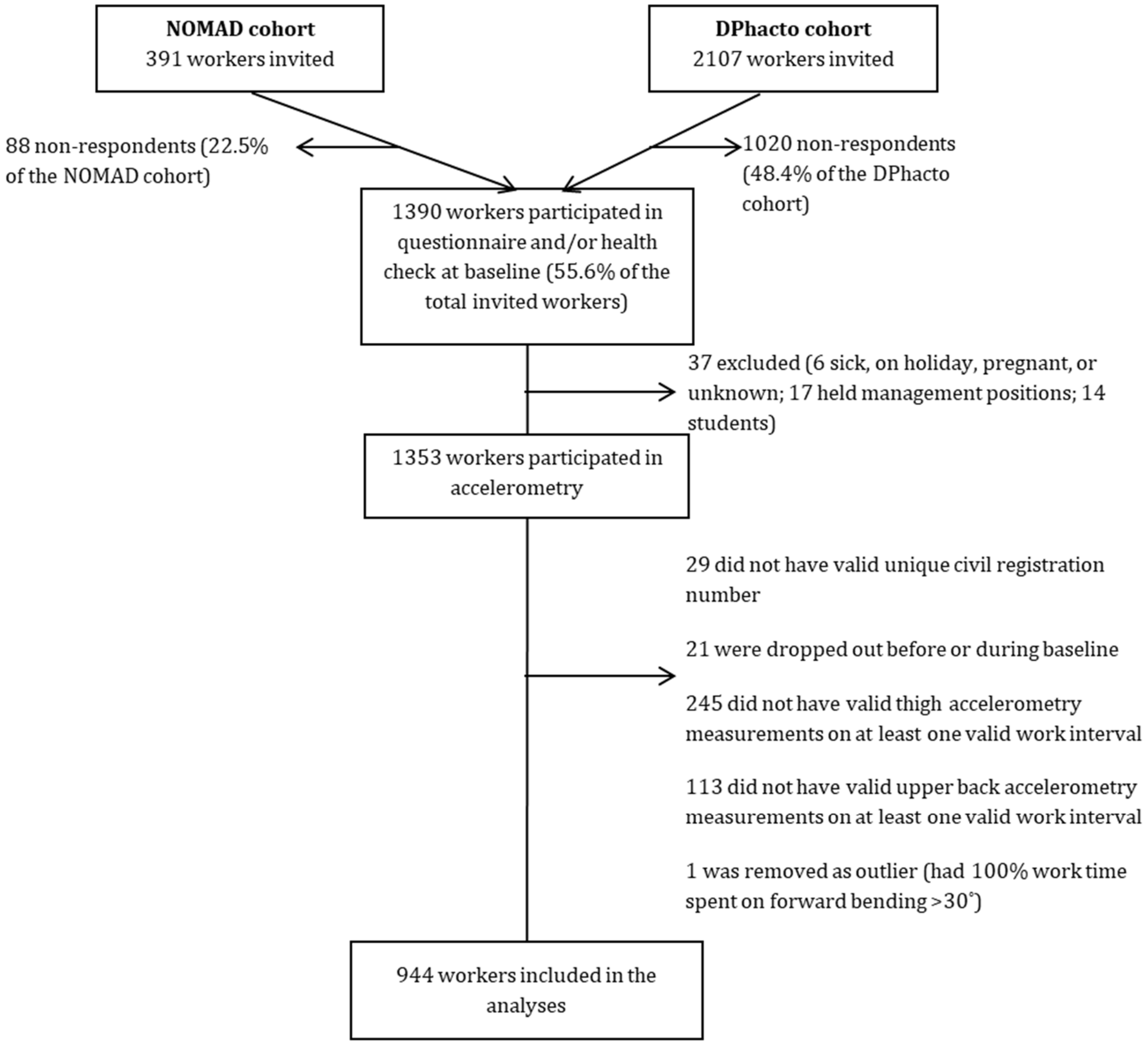

Appendix A. Participant flow.

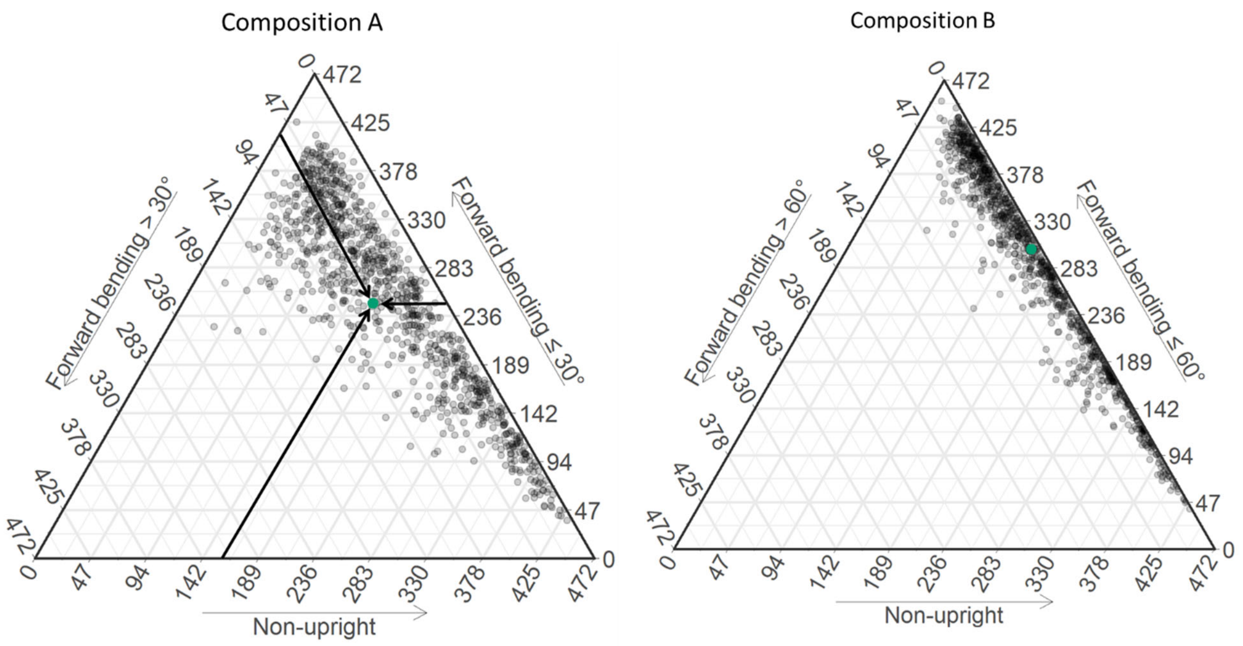

Appendix B= Ternary plot visualizing the distribution of the measured composition of the worktime per day (minutes) with forward bending of the back >30˚ (Composition A) and >60˚ (Composition B). The black dots are each worker's (in total 944 workers) composition while the green dot is the average composition of the study population. The arrows towards the average composition in the left ternary plot show an example of how to read data in a ternary plot. For example, the arrows indicate that the mean composition A is 40 minutes >30˚, 261 minutes ≤30˚ and 156 minutes in the non-upright position.

Appendix C. Regression estimates obtained from the Cox models to investigate the association between worktime with forward bending of the back >30˚ and >60˚ in an upright position, relative to worktime with forward bending ≤30˚ and ≤60˚, respectively, and prospective risk of long-term sickness absence.

	Dependent variable			
	**Long term sickness absence leave**			
	Model 1[a]		Model 2[b]	
**Variable**	**HR(95%CI)**	**P**	**HR(95%CI)**	**P**
Ilr_1	1.36(1.04-1.79)	0.03	1.25(1.01-1.54)	0.04
Ilr_2	0.73(0.57-0.93)	0.01	0.78(0.62-0.98)	0.03
Age	0.98(0.97-1.00)	0.04	0.99(0.97-1.00)	0.07
Men (Ref=Women)	0.52(0.39-0.69)	<0.001	0.52(0.39-0.70)	<0.001
BMI	1.00(0.97-1.03)	0.976	1.00(0.97-1.03)	0.91
Occupational Lift/carry duration	0.93(0.84-1.02)	0.13	0.93(0.84-1.03)	0.14
Blue collar (Ref=white-collar)	1.40(0.69-2.86)	0.35	1.41(0.69-2.88)	0.34

[a]Model 1: ilr based on forward bending composition A (>30˚)
[b]Model 2: ilr based on forward bending composition B (>60˚)
Case-wise deletion was used in the regression that resulted in removing 16 observations. Final sample used in the analysis was 928 and the number of events were 202. BMI=Body mass index.
